# Skin Absorption of Radionuclide and Hybrid Cleaning Solutions

**DOI:** 10.3390/toxics14080649

**Published:** 2026-07-23

**Authors:** Magdalena Długosz-Lisiecka, Agnieszka Adamus-Włodarczyk, Aleksandra Zymni, Teresa Jakubowska, Kamil Biały, Michał Biegała

**Affiliations:** 1 Institute of Applied Radiation Chemistry, Faculty of Chemistry, Lodz University of Technology Wróblewskiego 15, 90-924 Łódź, Poland; 2 Department of Medical Physics, Copernicus Memorial Hospital in Lodz Comprehensive Cancer Center and Traumatology, Pabianicka 62, 93-513 Łódź, Poland; 3 Department of Medical Imaging Technology, Medical University of Lodz, ul. Narutowicza 60, 90-136 Łódź, Poland; 4 The Central School of the State Fire Service in Czestochowa, ul. Sabinowska 62/64, 42-200 Częstochowa, Poland

**Keywords:** radioactivity contamination, CBRN, radiological hazard, hybrid method of decontamination, radiological emergency, skin decontamination, crisis response

## Abstract

This study primarily aimed to evaluate how the chemical form and carrier medium of radionuclide contamination influence the effectiveness of skin decontamination procedures. In addition, the study sought to identify decontamination strategies that align with recommended practices while reducing reliance on intensive mechanical cleaning methods. Although skin damage was not directly evaluated, the findings provide valuable information for improving the safety of decontamination procedures used by personnel handling radioactive materials and by emergency responders involved in radiological and CBRN (Chemical, Biological, Radiological, and Nuclear) incidents. Accidental spills of radiopharmaceuticals in laboratories and medical facilities, as well as contamination associated with uranium mining, fuel-cycle operations, spent fuel management, and the decommissioning of nuclear facilities, may involve radioactive isotopes present in a variety of chemical forms and solutions. Fresh porcine skin was used as an experimental model, and skin temperature was maintained at 37 °C to simulate physiological conditions. Europium-152 (^152^Eu) was selected as the model radionuclide. Deionized water, concentrated nitric acid (HNO_3_), saturated sodium hydroxide (NaOH) solution, and ethanol were used as carrier media representing different chemical environments of ^152^Eu contamination. To simulate realistic contamination scenarios, contaminating solutions were allowed to dry on the skin surface before decontamination. The influence of contaminant chemistry, carrier medium, and drying conditions on radionuclide penetration and subsequent decontamination effectiveness was investigated. Particular attention was given to the extent to which different physicochemical forms of contamination affected radionuclide removal from the skin. The results demonstrated that the chemical form of the contaminant and the drying conditions were key factors determining decontamination efficiency. Among the tested methods, a decontamination kit consisting of a soap-based solution, the complexing agent DTPA, and an absorbent non-woven swab achieved the highest radionuclide removal efficiency. These findings indicate that successful radionuclide decontamination depends strongly on the physicochemical properties of the contaminant. The combined use of a complexing agent, detergent-based formulation, and absorbent material can significantly enhance radionuclide removal from contaminated skin surfaces. Furthermore, the study highlights the importance of considering contaminant chemistry when developing effective and safe decontamination protocols for radiological and CBRN incidents.

## 1. Introduction

Decontamination of radioactive contamination from the skin is a critical component of radiological protection in occupational, medical, and emergency settings. The skin constitutes the first barrier against external contaminants; however, when radioactive materials are deposited on its surface—particularly in the presence of cuts, abrasions, or chemically aggressive solutions—there is a risk of deeper penetration and subsequent internal contamination. Such incidents may occur during radiopharmaceutical preparation and administration, in isotope laboratories, during radioactive waste processing, in nuclear fuel cycle facilities, during decommissioning activities, and in CBRN response scenarios [1,2,3,4,5].

A wide range of radionuclides may be involved in contamination events, depending on the working environment. In nuclear medicine and radiopharmacy, common examples include technetium-99m, iodine-131, fluorine-18, lutetium-177, and yttrium-90. In the nuclear industry, waste management, and decommissioning operations, workers may be exposed to radionuclides such as cesium-137, cobalt-60, strontium-90, uranium isotopes, plutonium isotopes, and other transuranic elements [1,2,3,4,5,6]. These radionuclides may occur in different chemical and physical forms, including aqueous solutions, acidic or alkaline media, colloids, and particulates, all of which strongly influence their adherence to the skin, penetration behavior, and the effectiveness of subsequent decontamination [7,8].

The severity of contamination is further influenced by the chemical nature of the contacting solution. Strong acids and bases may disrupt the epidermal barrier and facilitate deeper transport of radioactive species into the skin. Acidic solutions typically induce coagulative necrosis and tissue dehydration, whereas concentrated alkaline solutions may cause liquefactive necrosis, lipid saponification, and deeper tissue injury, thereby increasing the difficulty of decontamination [9,10,11]. Accordingly, the effectiveness of skin decontamination depends not only on the radionuclide itself, but also on its chemical form, the contamination matrix, exposure time, and the condition of the skin.

Several decontamination approaches have been investigated to improve radionuclide removal from skin and wounds. These include gentle washing protocols, detergents, chelating agents such as DTPA, adsorbent-based formulations, dermabrasive creams, and hydrogel systems designed to bind radionuclides while limiting their spread across the contaminated surface [12,13,14,15,16,17,18,19,20]. Previous studies have shown that immediate washing may substantially reduce surface contamination, whereas excessive scrubbing can damage the skin barrier and potentially increase radionuclide penetration [14]. Advanced formulations containing complexing agents or adsorbents may further enhance removal efficiency, particularly for metallic radionuclides; however, their performance depends on both the physicochemical properties of the contaminant and the application conditions [12,13,16,17].

From a toxicological and occupational health perspective, rapid and effective skin decontamination is important because it may reduce both local tissue exposure and the risk of further dermal penetration followed by internal uptake. However, comparative studies performed under standardized and physiologically relevant conditions remain limited, particularly for direct head-to-head evaluation of liquid, emulsion, and hydrogel-based formulations intended for radiometal removal from skin. Such comparative assessment is needed to identify formulations that maximize radionuclide removal while minimizing additional mechanical or chemical stress to the skin barrier.

In the present study, we evaluated the decontamination efficiency of selected liquid, emulsion, and hydrogel formulations applied to radionuclide-contaminated skin under controlled experimental conditions. Fresh porcine skin was used as an ex vivo model because of its structural similarity to human skin, and contamination was performed at 37 °C to approximate physiological conditions. The study was designed to compare the removal efficiency of formulations containing detergents, chelating agents, extractants, antioxidants, and adsorbents after contamination with europium-152. Europium-152 was selected as a model radiometal because its behavior is suitable for comparative assessment of decontamination performance in a controlled experimental system. Europium is a trivalent lanthanide whose coordination chemistry resembles that of many radiometals relevant to contamination scenarios. Furthermore, Eu-152 is frequently used as a surrogate radionuclide in radiochemical investigations and provides suitable gamma emissions for reliable quantitative measurements.

The novelty of this study lies in the systematic, side-by-side quantitative comparison of multicomponent decontamination systems under identical contamination conditions using a controlled ex vivo porcine skin model and radiotracer-based gamma spectrometry.

The specific objective of this work was to evaluate and compare the effectiveness of several decontamination formulations for removing radionuclide contamination from skin. Although the present study was focused on decontamination performance rather than direct biological endpoints such as tissue injury or healing, its findings remain relevant to toxicological risk mitigation because rapid and effective removal of radionuclide contamination may reduce both local tissue exposure and the likelihood of further systemic uptake. These results may contribute to the optimization of practical skin decontamination procedures relevant to laboratory incidents, medical isotope handling, nuclear industry operations, and radiological emergency response.

## 2. Materials and Methods

### 2.1. Tested Skin

Fresh porcine skin was used as an ex vivo model of human skin. The skin was obtained from a local meat-processing facility in Łódź, Poland, and was not subjected to tanning or any chemical or mechanical treatment in order to preserve its native structure, collagen organization, and endogenous lipid–protein composition. The ex vivo porcine skin model was selected because of its well-documented anatomical and physicochemical similarities to human skin, making it a suitable model for investigating radionuclide skin penetration and the effectiveness of decontamination procedures.

Prior to the experiments, the skin was cut into samples of uniform dimensions, and the contaminated area was standardized to 4 cm × 4 cm. Skin specimens were visually inspected before contamination, and samples exhibiting visible defects, lesions, or structural irregularities were excluded from the study. To ensure reproducibility and comparability of the results, all experimental parameters, including contaminant volume, contact time, decontamination procedure, and environmental conditions, were standardized throughout the study. Each decontamination procedure consisted of a single wiping pass using gentle manual pressure. To minimize operator-dependent variability, all decontamination procedures were performed by the same operator following a standardized wiping protocol.

Throughout the contamination and decontamination procedures, skin temperature was maintained at 37 °C to mimic physiological conditions.

### 2.2. Isotopic Tracer

The contaminating media consisted of selected chemical solutions labeled with europium-152 (^152^Eu) used as a radiotracer. Europium-152 was supplied by Eurostandard CZ as EuCl_3_ in 3 g HCl L^−1^. All radionuclide handling and sample preparation were performed in a Class III radiochemical laboratory (licensed by the President of the Polish National Atomic Energy Agency, PAA).

The stock solution consisted of a mono-isotopic aqueous ^152^Eu solution with an activity concentration of 1059.0 ± 0.6 Bq mL^−1^ at the reference date [2,3].

For preparation of the contaminating solutions, 0.1 mL of the ^152^Eu stock solution was added to 20 mL of each selected medium: deionized water (Milli-Q water purification system, Merck Millipore, Darmstadt, Germany), concentrated nitric acid (HNO_3_, (65%, analytical grade Chempur, Piekary Śląskie, Poland), saturated sodium hydroxide solution (NaOH, (analytical grade, Avantor Performance Materials Poland S.A., Gliwice, Poland), and ethanol (96%, analytical grade, Chempur, Piekary Śląskie, Poland). The selected media represented chemically diverse contamination scenarios, allowing assessment of how solution chemistry influences radionuclide retention on the skin and subsequent decontamination efficiency.

A volume of 0.10 mL of contaminating solution was applied to the defined skin area (4 cm × 4 cm). This corresponded to approximately 6.25 µL cm^−2^. Samples were left for approximately 30 min before decontamination to enable evaporation of the carrier medium and formation of dried contamination on the skin surface. The study focused on dried contamination because it is generally associated with stronger radionuclide retention and reduced decontamination efficiency compared with freshly deposited contaminants, thereby representing a more demanding and operationally relevant exposure scenario.

This study provides a systematic comparison of multicomponent decontamination systems combining surfactants, chelating agents, and sorbents, evaluated under strictly standardized contamination conditions on ex vivo porcine skin, enabling direct quantitative assessment of radionuclide removal efficiency using gamma spectrometry.

**Figure 1:**
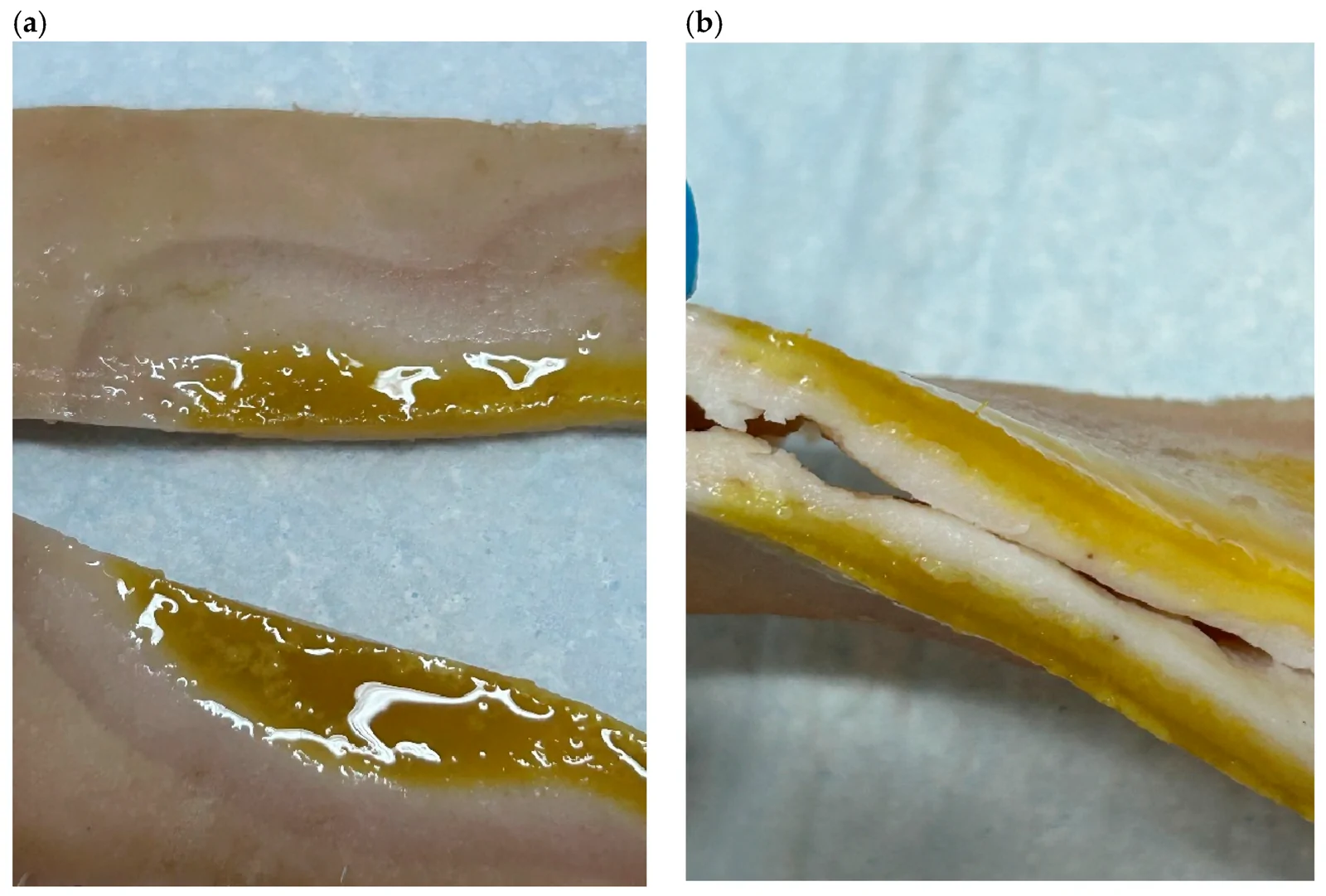
(**a**) Photograph of skin exposed to nitric acid (concentrated), which caused a wound within 30 min of being introduced to the surface. (**b**) Photograph of a cross-section of skin exposed to acid. The photo indicates the acid penetrating deep layers of the skin, introducing radionuclide contamination into deeper skin layers.

A few seconds of contact with concentrated nitric acid may cause superficial skin burns, whereas prolonged exposure can result in severe full-thickness tissue injury. Nitric acid binds free electrons in proteins, leading to protein denaturation, while the accompanying exothermic reaction may additionally contribute to thermal tissue damage. Concentrated nitric acid (V) produces a characteristic yellow discoloration of the burn eschar (Figure 1a,b). Consequently, the time elapsed between contamination and decontamination is a critical factor influencing both the extent of skin damage and the effectiveness of radionuclide removal. As contamination dries and chemical injury progresses, radionuclides may become more strongly associated with the skin surface and penetrate deeper into damaged tissue, making decontamination increasingly difficult. For this reason, the present study focused on dried contamination and samples were left for approximately 30 min before decontamination to simulate a challenging yet operationally relevant scenario.

### 2.3. Decontamination Material

During the experiment, various decontamination materials were used to remove radioactive contamination from the skin. Decontamination materials were prepared using the following materials, in different configurations (Table 1):

When radionuclides come into contact with skin, factors such as skin permeability especially in wound, or irritation regions, the chemical form of the radionuclide, and the presence of organic or inorganic entities are significant in the absorption process; therefore, decontamination effectiveness depends on several factors, which should be implemented in routine decontamination procedure.

### 2.4. Decontamination Techniques

To evaluate not only the influence of contaminant chemistry but also the role of the decontamination applicator on radionuclide removal efficiency, several types of wiping materials were selected. Tissue swabs, hydrogels, and microfiber cloths were chosen because they differ substantially in their physicochemical properties, including surface area development, porosity, absorbency, fluid retention capacity, and contact characteristics with the skin surface. These differences may affect both the uptake of dissolved radionuclides and the mechanical removal of contaminants from the skin. The materials were impregnated with the tested decontamination formulations and used in a series of standardized decontamination procedures. The decontamination protocols were organized into experimental series designated A–N, differing in both the type of decontamination material and the formulation applied.

Table 1 shows the removed portion of isotope from skin from different chemical forms of ^152^Eu radionuclide contaminations [%].

The tested decontamination formulations consisted of deionized water combined with selected sorbents, viscosity-modifying agents, surfactants, and radionuclide-complexing compounds. The investigated additives included activated carbon, hydrogel, laponite, detergent (soap), and selected chelating agents. The use of a surfactant was intended to reduce surface tension, thereby improving wetting of the contaminated skin surface and facilitating contact between the contaminant and active decontamination components.

Chelating agents capable of binding metal ions were incorporated into the formulations, including DTPA (diethylenetriaminepentaacetic acid; 97%, Lancaster, England), HDEHP (di(2-ethylhexyl) phosphoric acid; 97%, Sigma-Aldrich, St. Louis, MO, USA), and TIOA (trioctylamine; technical grade, Sigma-Aldrich, St. Louis, MO, USA). The quantities of all additives used in the individual formulations are presented in Table 1.

DTPA was applied in its free-acid form. The required amount of DTPA powder was dispersed directly in deionized water together with the remaining formulation components immediately before use. No neutralization or conversion to the sodium salt was performed. Although complete dissolution of DTPA cannot be guaranteed under all conditions, the presence of DTPA in dissolved and/or suspended form contributed to radionuclide removal through complexation of released metal ions. Since all formulations were prepared and evaluated using the same protocol, the comparative assessment of their decontamination performance remains valid.

All formulations were mixed until a homogeneous suspension or solution was obtained and subsequently applied to the selected wiping materials as specified in Table 1.

Gamma-ray spectrometry was performed using a high-purity germanium (HPGe) detector (Canberra, relative efficiency 30%). Measurements were carried out for both contaminated skin samples and the decontamination materials used during the cleaning procedure in order to quantify the distribution of ^152^Eu activity and evaluate decontamination efficiency.

Each decontamination procedure generated two samples for analysis: (i) the wiping material containing the radionuclide fraction removed from the skin and (ii) the skin sample containing residual radionuclide activity remaining after decontamination. Both sample types were measured using identical detector geometry, allowing direct comparison of their activities and determination of decontamination efficiency.

The activity of ^152^Eu was determined using its characteristic gamma-ray emissions at approximately 40.1 and 121.8 keV. Spectra were processed using dedicated gamma-spectrometry software, and sample activities were calculated from net peak areas following background subtraction and detector-efficiency correction.

Decontamination efficiency was evaluated by comparing the activity remaining on the skin surface after decontamination with the activity recovered on the corresponding decontamination material. The percentage of radionuclide removed from the skin was calculated using gamma-spectrometric measurements of both residual activity remaining on the skin and activity transferred to the decontamination material.

Each measurement was associated with a counting uncertainty derived from counting statistics. Error bars represent the uncertainty of individual gamma-spectrometric determinations and therefore reflect measurement precision rather than variability between independent experimental replicates.

## 3. Results

The composition of the decontamination formulations (A–N) is provided in the Section 2. Residual ^152^Eu activity was detected on all contaminated skin samples following the standardized contamination period, confirming retention of the radionuclide on the skin surface and/or within superficial skin layers.

The physicochemical form of contamination was identified as the primary factor influencing decontamination efficiency. Substantial differences were observed between the tested contamination media. Following a single decontamination cycle, the mean fraction of ^152^Eu removed from the skin reached 91.4% for ethanol-based contamination and 88.8% for alkaline contamination (saturated NaOH solution), whereas lower removal efficiencies were observed for aqueous contamination (75.9%) and particularly for acidic contamination (51.7%).

The markedly reduced decontamination efficiency observed for nitric acid-based contamination indicates that acidic conditions represent the most challenging contamination scenario. This effect may be related to acid-induced alterations of the skin structure, including protein denaturation and superficial tissue damage, which can promote stronger radionuclide retention and hinder subsequent removal from the skin surface.

Across all contamination media, the highest radionuclide removal efficiencies were generally achieved using formulations containing a detergent and a complexing agent. Formulations lacking either a surfactant or a chelating/extracting component consistently exhibited lower performance. The addition of DTPA resulted in a substantial improvement in radionuclide removal, particularly when combined with detergent-containing formulations. This observation confirms the importance of complexation processes in facilitating the mobilization and transfer of radionuclides from contaminated skin to the decontamination material.

The type of wiping material also affected decontamination performance. Microfiber-based applicators generally provided higher removal efficiencies than cellulose-based swabs, especially when used in combination with detergent-containing formulations. This improvement can be attributed to the larger effective contact area, higher sorption capacity, and enhanced interaction of microfiber materials with surface irregularities of the skin.

Hydrogel- and laponite-based systems demonstrated intermediate performance, with their effectiveness depending on both the composition of active additives and the type of wiping material employed. Although these materials provided measurable radionuclide removal, their overall performance was generally lower than that achieved by formulations combining detergent and DTPA.

**Figure 2:**
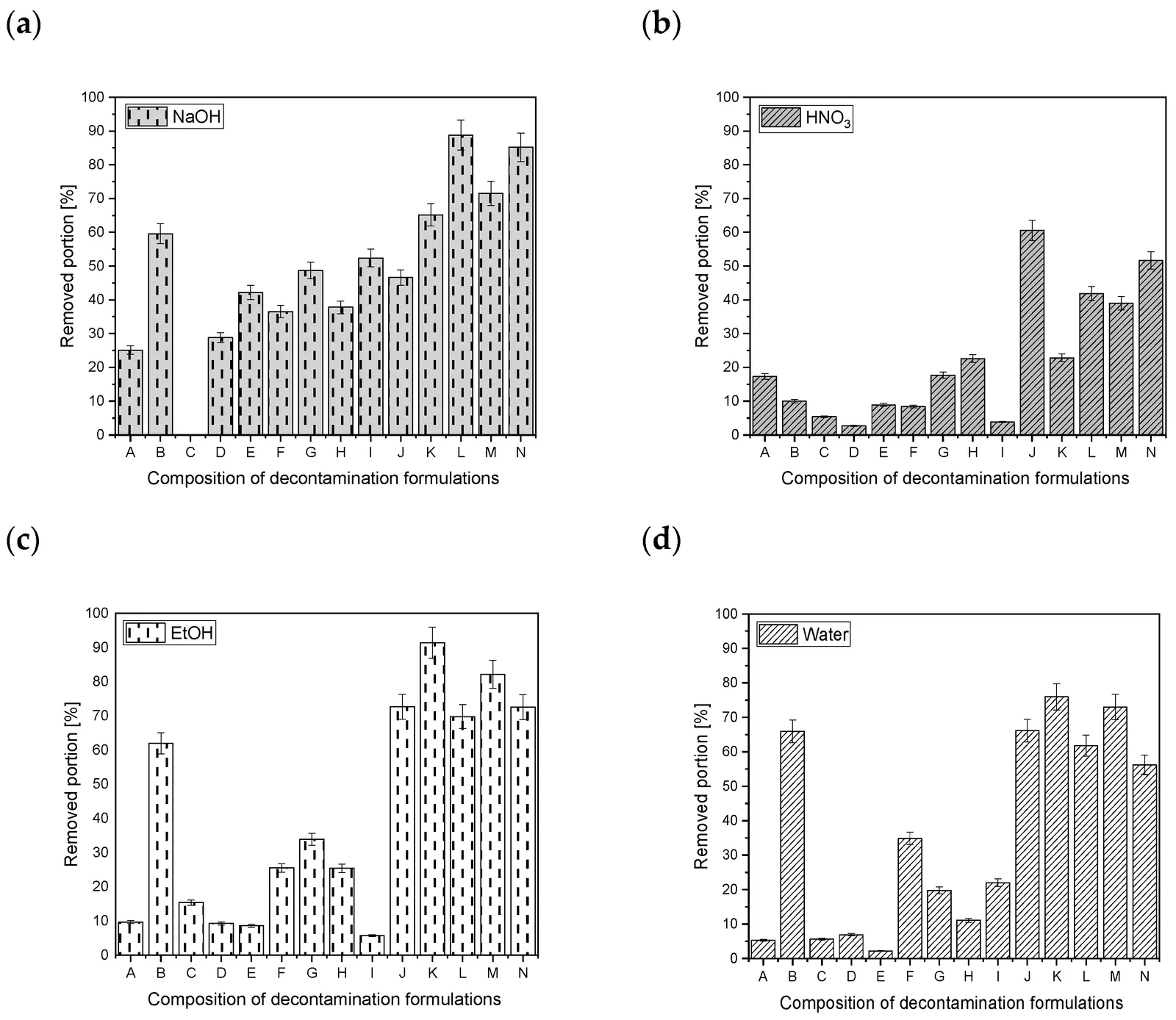
Percentage of ^152^Eu removed from skin for different chemical forms of contamination: (**a**) saturated NaOH solution, (**b**) concentrated HNO_3_, (**c**) ethanol, and (**d**) deionized water [%]. Error bars represent the counting uncertainties associated with individual gamma-spectrometric measurements.

The percentage of ^152^Eu removed from the skin for the different physicochemical forms of contamination is presented in Figure 2.

The percentage of radionuclide removed from the skin was calculated using gamma-spectrometric measurements of both residual activity remaining on the skin and activity transferred to the decontamination material. Error bars shown in Figure 2 represent the counting uncertainties associated with individual gamma-spectrometric measurements. These uncertainties originate from counting statistics and therefore reflect measurement precision rather than variability between independent experimental replicates.

Despite the use of a single decontamination cycle and the application of dried contamination conditions, several formulations achieved radionuclide removal efficiencies exceeding 80–90%. Because dried contamination is generally associated with stronger binding to the skin surface and reduced decontamination effectiveness compared with freshly deposited contamination, these findings indicate that appropriately designed multicomponent formulations can effectively remove radionuclides even under challenging conditions.

Overall, the results demonstrate that the physicochemical form of contamination is a critical determinant of decontamination effectiveness. While the composition of the decontamination formulation and the properties of the wiping material significantly influenced radionuclide removal, the highest efficiencies were consistently achieved with formulations combining a detergent, the complexing agent DTPA, and a high-surface-area absorbent material. These findings highlight the importance of considering both contaminant chemistry and decontamination system design when developing effective skin decontamination strategies for radiological and CBRN incidents.

## 4. Discussion

This study was designed as a comparative physicochemical evaluation of radionuclide skin decontamination and is limited to a single radionuclide model (^152^Eu) and an ex vivo porcine skin system. Consequently, the results should not be interpreted as a direct measure of clinical performance. Nevertheless, the use of physiologically relevant skin tissue under controlled experimental conditions enabled systematic assessment of factors governing radionuclide retention and removal from contaminated skin surfaces.

The most important finding of the present study is that the physicochemical form of contamination was a major determinant of decontamination efficiency. The observed differences between acidic, alkaline, aqueous, and ethanol-based contamination media were substantially greater than the differences observed among individual decontamination formulations within the same contamination category. In particular, the approximately 40 percentage-point difference between acidic contamination (51.7%) and ethanol-based contamination (91.4%) indicates that contaminant chemistry may exert a greater influence on radionuclide removal than the choice of decontamination formulation itself. These findings emphasize that effective decontamination strategies should be selected not only according to the radionuclide involved but also according to the chemical environment in which contamination occurs.

The results further demonstrate that radionuclide removal is governed by a combination of mechanical, sorptive, and chemical mechanisms. The highest decontamination efficiencies were consistently achieved using formulations containing both a detergent and the complexing agent DTPA. The effectiveness of these systems can be explained by complementary mechanisms of action. Detergents reduce surface tension, improve wetting of contaminated skin, and facilitate contact between active formulation components and the contaminant. At the same time, DTPA forms highly stable water-soluble complexes with trivalent radiometals such as europium, thereby promoting their transfer from the skin surface to the decontamination material and reducing the likelihood of re-adsorption [21,22,23]. The effectiveness of this process is strongly dependent on the coordination chemistry, ionic charge, and complex stability of the target metal ion, which explains the high affinity of DTPA toward trivalent lanthanides such as europium [24]. The superior performance of detergent–DTPA formulations observed in this study supports previous findings that multicomponent decontamination systems are generally more effective than single-component approaches for the removal of metallic radionuclides [25].

An additional observation concerns the role of the decontamination material itself. Although cellulose-based swabs effectively absorbed contamination, their lower liquid-retention capacity may facilitate partial release of absorbed contaminants back onto the skin surface during wiping. In contrast, microfiber-based materials consistently demonstrated higher decontamination efficiencies. This effect is likely related to their larger effective surface area, capillary network, and superior sorptive properties, which promote contaminant uptake while limiting redistribution during the wiping process [26]. The results indicate that optimization of applicator materials may contribute significantly to overall decontamination performance and should be considered alongside the chemical formulation.

The pronounced differences observed between contamination media highlight the importance of contaminant–skin interactions. Acidic contamination represented the most challenging decontamination scenario. A contributing factor may have been the experimental design, in which decontamination was intentionally delayed for approximately 30 min to allow contaminant drying. Dried contamination is generally more difficult to remove than freshly deposited contamination and better reflects operational conditions that may occur during radiological emergencies. Under such conditions, nitric acid may alter superficial skin structures through processes such as protein denaturation and disruption of barrier properties, potentially promoting stronger radionuclide retention. Although skin damage was not directly assessed in the present study, the markedly lower removal efficiencies observed for HNO_3_-based contamination suggest that chemically aggressive contamination media may significantly reduce subsequent decontamination effectiveness.

The importance of contamination chemistry observed in the present study is consistent with previous investigations involving radionuclides such as ^137^Cs and ^60^Co, where both skin penetration and decontamination effectiveness depended strongly on radionuclide form and exposure conditions [27]. These findings are also in agreement with current radiological protection recommendations, which recognize that radionuclide-specific physicochemical properties should be considered when selecting contamination management and decorporation procedures [28].

## 5. Conclusions

This study demonstrated that the physicochemical form of contamination is a key determinant of radionuclide skin decontamination efficiency. Significant differences were observed between acidic, alkaline, aqueous, and ethanol-based contamination media, indicating that contaminant chemistry may have a greater influence on decontamination effectiveness than differences between individual decontamination formulations.

Among the investigated contamination scenarios, acidic contamination proved to be the most difficult to remove, whereas ethanol-based and alkaline contamination showed substantially higher decontamination efficiencies. These findings highlight the importance of considering the chemical environment of contamination when selecting or developing skin decontamination procedures for radiological and CBRN incidents.

The highest radionuclide removal efficiencies were consistently achieved using multicomponent formulations combining a detergent, the complexing agent DTPA, and highly absorbent wiping materials. The effectiveness of these systems can be attributed to the complementary action of surfactant-mediated contaminant mobilization, metal-ion complexation, and efficient contaminant uptake by the wiping material.

The study also demonstrated that the type of applicator material significantly influences decontamination performance. Microfiber-based materials generally outperformed cellulose-based swabs, suggesting that optimization of wiping materials may contribute substantially to overall decontamination effectiveness.

Despite the use of dried contamination and only a single decontamination cycle, several formulations achieved radionuclide removal efficiencies exceeding 80–90%. These findings indicate that appropriately designed multicomponent decontamination systems can provide effective radionuclide removal under realistic operational conditions.

Although the study was limited to ^152^Eu and an ex vivo porcine skin model, the results provide valuable information for the development of evidence-based skin decontamination strategies for personnel working with radioactive materials and for responders involved in radiological and CBRN emergencies.

## Figures and Tables

**Table 1:** Composition of decontamination formulations (A–N) used in the study.

	Cleaning Solution	Swob
A	water 10 mL, DTPA 1 g	100% cellulose
B	water 10 mL, charcoal 2.0 g, DTPA 0.5 g	100% cellulose
C	water 10 mL, charcoal 0.5 g, DTPA 0.5 g	100% cellulose
D	hydrogel 3.3 g, DTPA 0.2 g, charcoal 0.2 g	100% cellulose
E	laponite 2 g in 15 mL water	100% cellulose
F	laponite 2 g in 15 mL water, 0.2 g ascorbic acid	100% cellulose
G	laponite 2 g in 15 mL water, 0.2 g DTPA	100% cellulose
H	laponite 2 g in 15 mL water, 0.2 g HDEHP	100% cellulose
I	laponite 2 g in 15 mL water, 0.2 g TIOA	100% cellulose
J	water 10 mL, detergent 1 g	100% microfiber
K	water 10 mL, detergent 1 g	85% polyester + 15% polyamide
L	water 10 mL, detergent 1 g, DTPA 0.2 g	100% viscose
M	water 10 mL, detergent 1 g, DTPA 0.2 g	100% microfiber
N	water 10 mL, detergent 1 g, DTPA 0.2 g	85% polyester + 15% polyamide

## Data Availability

The raw data supporting the conclusions of this article will be made available by the authors on request.
